# Comprehensive Analysis Suggests Overlapping Expression of Rice ONAC Transcription Factors in Abiotic and Biotic Stress Responses

**DOI:** 10.3390/ijms16024306

**Published:** 2015-02-17

**Authors:** Lijun Sun, Lei Huang, Yongbo Hong, Huijuan Zhang, Fengming Song, Dayong Li

**Affiliations:** 1 National Key Laboratory for Rice Biology, Institute of Biotechnology, Zhejiang University, Hangzhou, Zhejiang 310029, China; E-Mails: slj.1226@163.com (L.S.); leihero2008@163.com (L.H.); yongbohong@126.com (Y.H.); zhanghj82@zju.edu.cn (H.Z.); fmsong@zju.edu.cn (F.S.); 2 Institute of Analytical Chemistry for Life Science, School of Public Health, Nantong University, Nantong 226019, China

**Keywords:** NAC transcription factors, overlapping expression, rice, abiotic and biotic stress

## Abstract

NAC (NAM/ATAF/CUC) transcription factors comprise a large plant-specific gene family that contains more than 149 members in rice. Extensive studies have revealed that NAC transcription factors not only play important roles in plant growth and development, but also have functions in regulation of responses to biotic and abiotic stresses. However, biological functions for most of the members in the NAC family remain unknown. In this study, microarray data analyses revealed that a total of 63 *ONAC* genes exhibited overlapping expression patterns in rice under various abiotic (salt, drought, and cold) and biotic (infection by fungal, bacterial, viral pathogens, and parasitic plants) stresses. Thirty-eight *ONAC* genes exhibited overlapping expression in response to any two abiotic stresses, among which 16 of 30 selected *ONAC* genes were upregulated in response to exogenous ABA. Sixty-five *ONAC* genes showed overlapping expression patterns in response to any two biotic stresses. Results from the present study suggested that members of the *ONAC* genes with overlapping expression pattern may have pleiotropic biological functions in regulation of defense response against different abiotic and biotic stresses, which provide clues for further functional analysis of the *ONAC* genes in stress tolerance and pathogen resistance.

## 1. Introduction

Plants are always subject to various types of abiotic (e.g., high-salinity, drought, and cold) and biotic (infection by fungal, bacterial and viral pathogens, and infestation by herbivores and parasitic plants) stresses under variable environmental conditions, and have developed a series of mechanisms at physiological, biochemical, and molecular levels to combat these stresses [1,2,3,4,5]. In response to abiotic and biotic stresses, plants often activate a battery of defense responses that include inducible expression of a set of stress-related genes, which are regulated directly or indirectly by different types of transcription factors. Previous studies have showed that dozens of transcription factors belonging to the ERF, MYB, WRKY, and bZIP families are involved in regulation of plant stress responses [6,7,8,9].

The NAC (NAM/ATAF/CUC) transcription factor family, as one of the largest plant-specific gene families, was first reported in 1997 [10]. NAC proteins are characterized with a conserved region at their *N*-terminal ends and a highly diverged *C*-terminus [11,12]. Genome-based bioinformatics analyses have showed that NAC proteins comprise a large family with more than 100 members in *Arabidopsis*, rice, tobacco, soybean, and *populus* [13,14,15,16,17,18]. However, only a few of them have been functionally characterized for their biological functions. To date, the members of the *NAC* gene family have been demonstrated to play important roles in diverse biological processes of plant growth and development, e.g., cell wall biosynthesis and senescence (for reviews, see [19,20]).

NAC transcription factors have been also demonstrated to play important roles in regulating plant abiotic and biotic stress responses. Transcriptional profiling analysis revealed that at least 33 *NAC* genes were responsible to abiotic stress, including high salt in *Arabidopsis* [21]. It was further showed that expression of *Arabidopsis ANAC019*, *ANAC055*, and *ANAC*0*72*/*RD26* was induced by different abiotic stresses including drought and high salt, and overexpression of *ANAC019*, *ANAC055*, *ANAC072*/*RD26*, or *ATAF1* significantly increased drought tolerance [22,23]. Furthermore, transgenic Arabidopsis plants overexpressing *ANAC072*/*RD26* were highly sensitive to ABA [24]. In rice, overexpression of stress-responsive *SNAC1* or *SNAC2* (*OsNAC6*) enhanced drought and salinity tolerance [25,26,27,28], and the *SNAC1*-overexpressing transgenic rice showed increasing drought tolerance in the field under severe drought stress conditions at the reproductive stage without any phenotypic changes or adverse effect on yields [25]. OsNAC5 as a transcriptional activator interacts with OsNAC6 and SNAC1 and overexpression of *OsNAC5* resulted in improved tolerance to high salt as compared to control plants [29]. Similarly, overexpression of *OsNAC10* (*ONAC122*) or *OsNAC045* in rice increased drought and salt tolerance [30,31]. Furthermore, chickpea *NAC* genes such as *CarNAC1*, *CarNAC3*, and *CarNAC5* and tomato *NAC* genes like *SlNAC1*, *SlNAM1*, and *SlNAC2* were also reported to be involved in abiotic stress responses [32,33,34,35,36].

Accumulating evidence has implied that the *NAC* genes play important roles in plant defense response against pathogen infection. The Arabidopsis *ATAF1* and *ATAF2* genes have been demonstrated through knockout or overexpression approaches to be negative regulators of defense response against different pathogens, e.g., *Botrytis cinerea*, *Pseudomonas syringae* pv. *tomato*, *Alternaria brassicicola*, or *Fusarium oxysporum* [23,37,38]. Barley *HvNAC6*, an *ATAF1* homologous gene, was shown to contribute to penetration resistance against *Blumeria graminis* f. sp. *hordei* [39]. In rice, three *ONAC* genes have been demonstrated to have function in disease resistance response. *OsNAC6* was induced by *Magnaporthe grisea*, the causal agent of blast disease, and overexpression of *OsNAC6* in rice resulted in improved blast resistance [28]. *OsNAC4* was shown to be involved in regulation of hypersensitive cell death because overexpression of *OsNAC4* led to extensive HR cell death, while knockdown of *OsNAC4* suppressed HR cell death in response to an avirulent bacterial pathogen [40]. *RIM1* (*ONAC054*) was revealed to be a specific *NAC* gene that negatively regulates defense response against rice dwarf virus (RDV) [41]. A Tos17-inserted mutant line showed no typical disease symptoms upon RDV infection but remained susceptible to other viral pathogens [41]. Other *NAC* genes such as *Arabidopsis TIP*, wheat *GRAB1*/*GRAB2*, and tomato *SINAC1* have also been reported to be involved in plant–virus interactions, probably through interacting with virus proteins [42,43,44]. It was also found that pepper *CaNAC1*, wheat *TaNAC4*, and soybean *GmNAC6* are pathogen responsible, suggesting their possible involvement in defense response to pathogen infection [45,46,47]; however, further functional characterization is required for these pathogen-responsive *NAC* genes to clarify their function in plant disease resistance response.

Although several *ONAC* genes such as *SNAC1*, *OsNAC6*, *OsNAC5*, *OsNAC10*, and *OsNAC4* have been characterized functionally in rice, the biological functions of most members in this family remain unknown. To extend our understanding of the biological function of the rice NAC family, we performed overlapping expression analysis using public microarray data and identified a total of 63 *ONAC* (following the nomenclature as suggested by Fang *et al.* [14]) genes that showed overlapping expression patterns under different stresses. The phylogenetic relationships suggested that the *ONAC* genes showing overlapping expression patterns under stress conditions can be classed into 10 groups. We also isolated 15 putative stress-responsive *ONAC* genes and analyzed their expression patterns in response to different abiotic and biotic stresses. Our results suggested that some members of the rice ONAC family may have overlapping functions in regulation of defense response against different abiotic and biotic stresses, and also provided clues for further functional analysis of *ONAC* genes in stress tolerance and pathogen resistance.

## 2. Results

### 2.1. Inducibility and Overlapping Expression of ONAC Genes in Response to Abiotic Stresses

**Figure 1:**
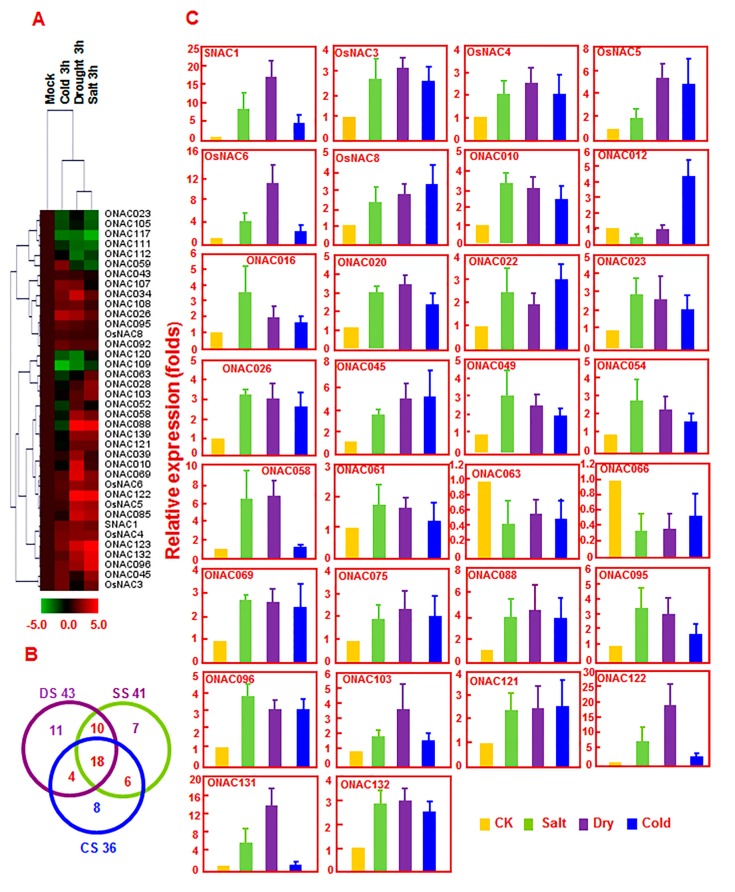
Overlapping expression of *ONAC* genes in response to various abiotic stress conditions. (**A**) Heat map of *ONAC* genes showing differential expression patterns under at least two abiotic stress conditions. The change values in treated samples *vs.* corresponding control, shown as (log_2_^(signal intensity in treatment/signal intensity in control)^ = log_2_^signal intensity in treatment^ − log_2_^signal intensity in control^), were used for Treeview (Supplementary file 2). The color scale of change values is shown at the bottom; (**B**) Venn diagram represents number of *ONAC* genes expressed commonly or specifically under given abiotic stress conditions (*t*-test *p* < 0.01); (**C**) Differential expression of 30 selected *ONAC* genes under drought, salt, and cold conditions, as analyzed by qPCR. Relative expression of each *ONAC* gene was calculated by comparison with corresponding control. Error bars represent standard errors of the means from three independent biological replicates.

**Figure 2:**
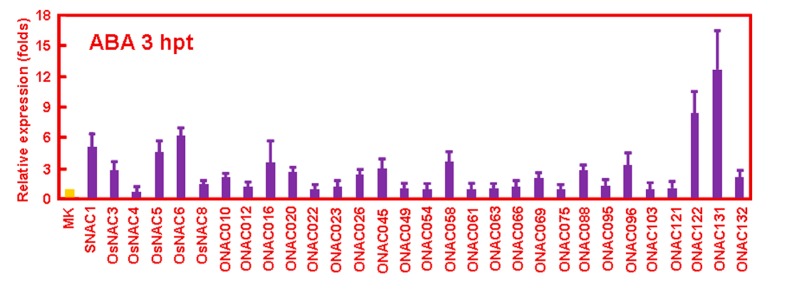
Differential expressions of 30 selected *ONAC* genes in response to ABA. Rice seedlings were treated with ABA for 3 h and expression of *ONAC* genes was analyzed by quantitative RT-PCR. Relative expression for each *ONAC* gene was calculated by comparison with corresponding control. Error bars represent standard errors of the means from three independent biological replicates.

Salt, drought, and cold are common stresses that cause adverse effects on the growth and development of plants and the productivity of crops [48]. To study the possible involvement of *ONAC* genes in response to various abiotic stresses, we mined microarray data (GSE6901) deposited in a public database [49] and performed systematic analysis for their expression patterns under salinity, drought, and cold conditions. Differential expression of the *ONAC* genes in rice seedlings treated with different abiotic stresses such as salinity, drought, and cold conditions were analyzed by comparison with mock-treated seedlings. For drought stress, 43 *ONAC* genes (33 upregulated and 10 downregulated) were differentially expressed in cv. IR64 seedlings after drought treatment for 3 h. For salt stress, 41 *ONAC* genes (31 upregulated and 10 downregulated) were differentially expressed in cv. IR64 seedlings after salt treatment for 3 h. For cold stress, 36 *ONAC* genes (27 upregulated and 9 downregulated) were differentially expressed in rice seedlings under cold stress (Supplementary file 1). Further analysis of the overlapping expression patterns identified a total of 38 *ONAC* genes that were differentially expressed under at least two abiotic stress conditions based on analysis of microarray data GSE6901. Among these, 15 *ONAC* genes (*SNAC1*, *OsNAC4*, *OSNAC5*, *OsNAC6*, *OsNAC8*, *ONAC026*, *ONAC034*, *ONAC069*, *ONAC092*, *ONAC095*, *ONAC096*, *ONAC108*, *ONAC122*, *ONAC123*, and *ONAC132*) were upregulated and 2 *ONAC* genes (*ONAC109* and *ONAC117*) were downregulated under drought, salt, and cold stress conditions (Figure 1A,B and Supplementary file 2). To confirm the results obtained from microarray data analysis, we analyzed by quantitative RT-PCR the expression levels of 30 selected *ONAC* genes in response to drought, salt, and cold stresses and the results showed that the expression patterns of these *ONAC* genes under drought, salt, and cold stress conditions were identical to the patterns obtained from the rice microarray data analysis (Figure 2C). However, the expression patterns for *ONAC016* and *ONAC023* were not identical (Figure 1C). Results from our overlapping expression and quantitative RT-PCR analyses are in accordance with previous studies in which genes such as *SNAC1*, *SNAC*2/*OsNAC*6, *OsNAC5*, *OsNAC10*, and *ONAC45* were induced by different stresses including drought, high salinity, and cold stresses [25,26,27,28,29,30,31].

ABA is a key mediator regulating plant response to a number of abiotic stresses such as drought, high salinity, and cold through activation of many stress-related genes [50]. On the other hand, ABA has also been demonstrated to play important roles in the regulation of plant disease resistance response [50,51]. Thus, we analyzed the expression of these representative *ONAC* genes in response to ABA treatment. Our data showed that the expression levels of these 16 *ONAC* genes, including *SNAC1*, *OsNAC3*, *OsNAC5*, *OsNAC6*, *ONAC010*, *ONAC016*, *ONAC020*, *ONAC026*, *ONAC045*, *ONAC058*, *ONAC069*, *ONAC088*, *ONAC095*, *ONAC122*, *ONAC131*, and *ONAC132* were upregulated in rice seedlings at 3 h after ABA treatment (Figure 2).

### 2.2. Inducibility and Overlapping Expression of ONAC Genes in Response to Biotic Stress

To explore possible involvement of *ONAC* genes in rice response to biotic stress, we analyzed the expression of the *ONAC* genes in rice seedlings after infection with different types of pathogens or infestation by parasitic plants.

**Figure 3:**
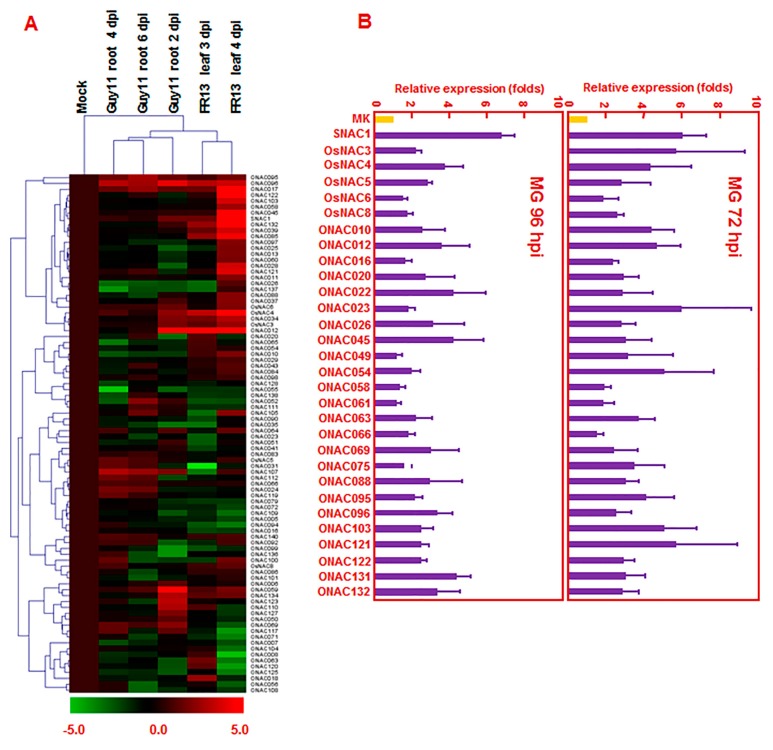
Differential expression of *ONAC* genes in response to infection by *M. oryzae.* (**A**) Heat map of *ONAC* genes showing differential expression patterns after leaf and root infection by *M. oryzae*. The change values in treated samples *vs.* corresponding control, shown as (log_2_^(signal intensity in treatment/signal intensity in control)^ = log_2_^signal intensity in treatment^ − log_2_^signal intensity in control^), were used for Treeview (Supplementary file 2). The color scale of change values is shown at the bottom; (**B**) Expression of 30 selected *ONAC* genes in response to infection by *M. oryzae*. Quantitative RT-PCR analysis was performed for samples of the leaves of 2-week-old Yuanfengzao collected at 72 and 96 h after inoculation with *M. oryzae*. Relative expression of each *ONAC* gene was calculated by comparison with corresponding control. Error bars represent standard errors of the means from three independent biological replicates.

Rice blast disease, caused by *M. oryzae*, is one of the most serious and widespread diseases of rice. Due to the agronomic importance of rice, it is important to understand the molecular mechanisms of disease resistance against *M. oryzae*. We first analyzed the expression patterns of *ONAC* genes by mining the microarray data GSE7256 (leaves of 14-day-old rice seedlings infected with a virulent strain FR13 of *M. grisea*) and GSE18361 (roots of rice seedlings infected with by strain Guy11 of *M. oryzae*) [52,53]. For leaf infection by strain FR13, a total of 75 *ONAC* genes that were differentially expressed at 3 and 4 days post-inoculation (DPI) were identified. Among them, 23 and 17 *ONAC* genes were upregulated and downregulated, respectively, at 3 DPI, whereas 42 and 21 *ONAC* genes were upregulated and downregulated, respectively, at 4 DPI (Supplementary file 1). Fifteen *ONAC* genes (*ONAC1*, *OsNAC3*, *OsNAC4*, *OsNAC8*, *ONAC010*, *ONAC012*, *ONAC017*, *ONAC034*, *ONAC039*, *ONAC059*, *ONAC085*, *ONAC095*, *ONAC096*, *ONAC132*, and *ONAC134*) were upregulated while 5 *ONAC* genes (*ONAC016*, *ONAC052*, *ONAC079*, *ONAC094*, and *ONAC138*) were downregulated at 3 and 4 DPI. For root infection by Guy11, a total of 64 *ONAC* genes were found to be differentially expressed at 2, 4, and 6 DPI. Among them, expression of 24, 19, and 19 *ONAC* genes at 2, 4, and 6 DPI, respectively, was upregulated, whereas expression of 17, 9, and 14 *ONAC* genes at 2, 4, and 6 DPI, respectively, was downregulated (Supplementary file 1). In total, 88 *ONAC* genes were differentially expressed after rice infection by *M. grisea* and 50 *ONAC* genes exhibited overlapping expression patterns between leaf and root infection by *M. grisea* (Figure 3A and Supplementary file 2). To confirm the results obtained from microarray data analysis, we analyzed by quantitative RT-PCR the expression levels of 30 selected *ONAC* genes in rice seedlings after leaf infection with *M. grisea* and our results showed that the expression patterns of these *ONAC* genes in response to infection of *M. grisea* at 3 and 4 DPI were similar to those obtained from microarray data analysis (Figure 3B). Among these *ONAC* genes that showed differential expression in response to *M. oryzae*, *OsNAC6* and *SNAC1*/*OsNAC19* were previously reported to be induced by *M. grisea* [28,54].

**Figure 4:**
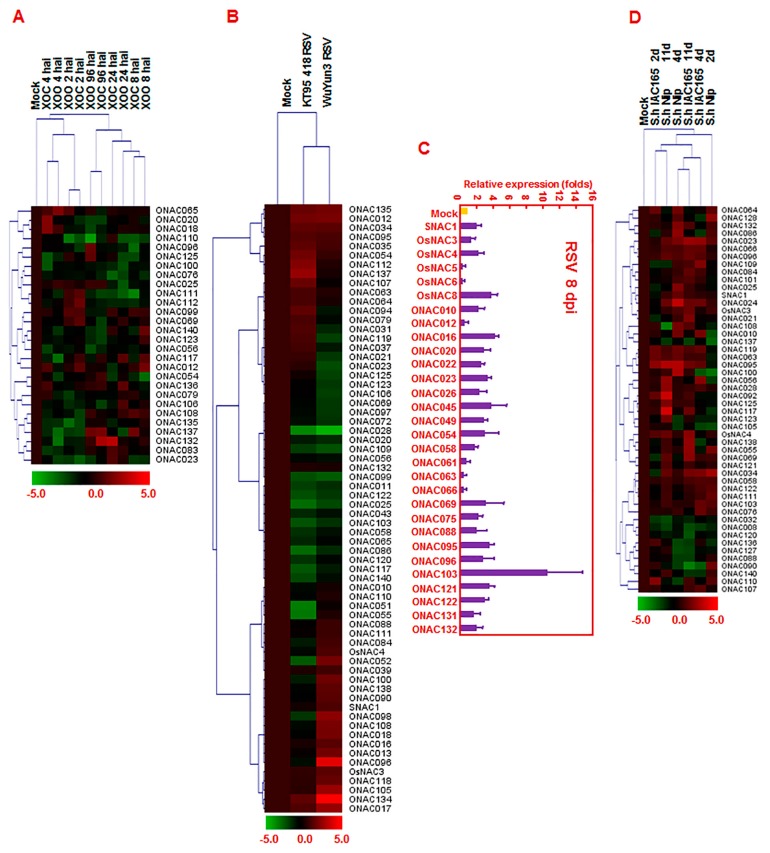
Differential expressions of *ONAC* genes in response to *Xoo*, *Xoc* and RSV. (**A**,**B**) Heat maps of *ONAC* genes showing differential expression patterns in rice seedlings after *Xoo* and *Xoc* infection and RSV infection, respectively. The change values in treated samples *vs.* corresponding control, shown as (log_2_^(signal intensity in treatment/signal intensity in control)^ = log_2_^signal intensity in treatment^ − log_2_^signal intensity in control^), were used for Treeview (Supplementary file 2). The color scale of change values is shown at the bottom; (**C**) Differential expression of 30 selected *ONAC* genes in rice after RSV infection; (**D**) Heat Map of *ONAC* genes showing differential expression patterns after infestation by *S. hemonthica*. Quantitative RT-PCR analysis was performed and relative expression for each *ONAC* gene was calculated by comparison with corresponding control. Error bars represent standard errors of the means from three independent biological replicates.

Bacterial blight caused by *Xanthomonas oryzae* pv. *oryzae* (*Xoo*) and bacterial leaf streak caused by *X. oryzae* pv. *oryzicola* (*Xoc*) are two major rice bacterial diseases with distinctive types of infection style. *Xoo* typically invades the xylem system, whereas *Xoc* generally infects and colonizes the mesophyll parenchyma. Previously, the abundance of transcripts in 14-day-old cv. Nipponbare seedlings at 2, 4, 8, 24, and 96 h after inoculation with *Xoo* strain PXO99A and *Xoc* strain BLS256 was analyzed (microarray data set GSE16793) to establish the global structure of susceptible responses to infection by *Xoo* and *Xoc* [55]. We used these data sets and analyzed the expression profiling of the *ONAC* genes’ response to infection with *Xoo* and *Xoc*. Our analyses showed that 5, 3, 7, 8, and 8 *ONAC* genes were upregulated and 6, 7, 2, 10, and 4 *ONAC* genes were downregulated at 2, 4, 8, 24, and 96 h, respectively, after inoculation with *Xoo* strains PXO99A; and that 10, 8, 9, 6, and 8 *ONAC* genes were upregulated and 6, 8, 5, 3, and 9 *ONAC* genes were downregulated at 2, 4, 8, 24, and 96 h, respectively, after inoculation with *Xoc* strain BLS256 (Supplementary file 1). Importantly, a total of 28 *ONAC* genes were found to exhibit overlapping expression patterns in rice plants after infection with *Xoo* and *Xoc*. Among these, 13 *ONAC* genes (*ONAC012*, *ONAC18*, *ONAC025*, *ONAC065*, *ONAC069*, *ONAC083*, *ONAC099*, *ONAC108*, *ONAC112*, *ONAC117*, *ONAC132*, *ONAC136*, and *ONAC140*) were upregulated and 10 *ONAC* genes (*ONAC020*, *ONAC023*, *ONAC056*, *ONAC076*, *ONAC100*, *ONAC110*, *ONAC111*, *ONAC125*, *ONAC135*, and *ONAC137*) were downregulated in both *Xoo*- and *Xoc*-infected rice plants (Figure 4A and Supplementary file 2).

Rice stripe disease, caused by rice stripe virus (RSV), which is transmitted by small brown planthoppers (*Laodelphax striatellus*), is one of the most serious viral diseases and causes severe yield loss in rice. Transcriptional profiling (microarray data set GSE11025) was performed using Affymetrix rice genome arrays and investigated the molecular differences in subspecies cv. WuYun3 and cv. KT95-418 in response to RSV infection (9-day old seedlings inoculated by exposing to approximately 100 viruliferous adults of *L. striatellus* for 2 days and then transplanted to an insect-free greenhouse at 25 ± 3 °C) [56]. Analyses of the expression patterns of *ONAC* genes in response to RSV using these microarray data resulted in identification of at least 27 *ONAC* genes that were upregulated and 16 genes were downregulated in cv. WuYun3, while 22 *ONAC* genes were upregulated and 19 genes were downregulated in cv. KT95-418 in response to RSV infection (Supplementary file 1). A total of 17 *ONAC* genes showed overlapping expression patterns in cv. WuYun3 and cv. KT95-418 plants after RSV infection. Among them, 7 *ONAC* genes (*ONAC012*, *ONAC17*, *ONAC034*, *ONAC035*, *ONAC095*, *ONAC134*, and *ONAC135*) were upregulated and 6 *ONAC* genes (*ONAC025*, *ONAC28*, *ONAC099*, *ONAC103*, *ONAC109*, and *ONAC122*) were downregulated (Figure 4B). We analyzed by quantitative RT-PCR the expression levels of 30 selected *ONAC* genes in rice seedlings after RSV infection and our results showed that the expression patterns of these *ONAC* genes in response to RSV were similar to those obtained from overlapping expression analysis using public microarray data (Figure 4C).

*Striga hermonthica* is a kind of root parasitic plant of rice and causes devastating loss of rice yield. Transcriptional profiling was performed to identify the possible molecular mechanism underlying susceptible and resistant responses of rice plants of different cultivars, where cv. Nipponbare is resistant and cv. IAC165 is susceptible, respectively, to infestation by *S. hermonthica* (three-week-old cv. Nipponbare and cv. IAC165 seedling infested with 25 mg of preconditioned and pregerminated seeds of *S. hermonthica* and root samples harvested at 2, 4, and 11 DPI) [57]. We mined the microarray data from the above-mentioned study and analyzed the expression patterns of *ONAC* genes in response to infestation by *S. hermonthica*. Our analyses revealed that 9, 24, and 30 *ONAC* genes were upregulated and 2, 9, and 9 *ONAC* genes were downregulated in cv. IAC165 at 2, 4, and 11 DPI, while 18, 28, and 32 *ONAC* genes were upregulated and 5, 7, and 5 *ONAC* genes were downregulated in cv. Nipponbare after infestation with *S. hermonthica* (Supplementary file 1). A total of 49 *ONAC* genes exhibited overlapping expression patterns in plants of cv. IAC165 and cv. Nipponbare after infection by *S. hermonthica* (Figure 4D and Supplementary file 2). Interestingly, the number of the *ONAC* genes with upregulated expression patterns was significantly more than that of the *ONAC* genes with downregulated expression patterns in response to *S. hermonthica* both in cv. IAC165 and cv. Nipponbare after infection by *S. hermonthica*, suggesting that most of the *ONAC* genes can be indeced by *S. hermonthica* infection.

**Figure 5:**
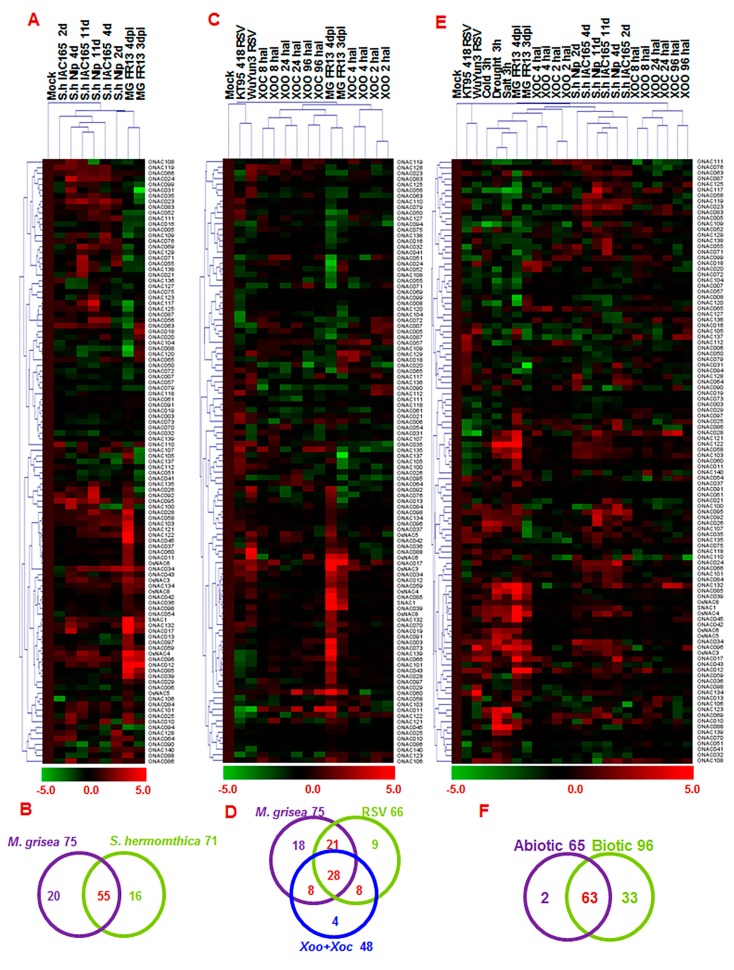
Overlapping expression of *ONAC* genes in response to various biotic and abiotic stresses. (**A**) Heat map of *ONAC* genes, showing differential expression patterns in response to *M. oryzae* and *S. hermonthica*; (**B**) Venn diagram represents number of *ONAC* genes expressed commonly or specifically in response to *M. oryzae* and *S. hermonthica* (*t*-test *p* < 0.01); (**C**) Heat map of *ONAC* genes showing differential expression patterns in response to *M. oryzae*, RSV, *Xoo*, and *Xoc*; (**D**) Venn diagram represents number of *ONAC* genes expressed commonly or specifically in response to *M. oryzae*, RSV, *Xoo*, and *Xoc* (*t*-test *p* < 0.01); (**E**) Heat map of *ONAC* genes showing differential expression patterns under abiotic (salt, drought, and cold) and biotic (infection by *M. oryzae*, *Xoo*, *Xoc*, or RSV, or infestation by *S. hermonthica*) stress; (**F**) Venn diagram represents number of *ONAC* genes expressed commonly or specifically in response to biotic and abiotic stresses (*t*-test *p* < 0.01). The change values (**A**,**C**,**E**) in treated samples *vs.* corresponding control, shown as (log_2_^(signal intensity in treatment/signal intensity in control)^ = log_2_^signal intensity in treatment^ − log_2_^signal intensity in control^), and the relative expression folds were calculated by 2^change values^. Both of them were used for Treeview (Supplementary file 2). The color scales of change values and relative expression folds were shown at the bottom.

### 2.3. Overlapping Expression of Different Groups of ONAC Genes in Response to Abiotic and Biotic Stresses

When comparing the expression patterns of the *ONAC* genes in response to different biotic stresses, we identified 51 *ONAC* genes with overlapping expression patterns in responses to infection by *M. oryzae* and to infestation by *S. hermonthica*. Among these, 22 *ONAC* genes were upregulated in response to *M. oryzae* and *S. hermonthica* (Figure 5A,B and Supplementary file 2). Similarly, 28 *ONAC* genes exhibited overlapping expression patterns in response to infection by different type of pathogens including *M. oryzae*, RSV, *Xoo*, and *Xoc* (Figure 5D), and at least 37 *ONAC* genes showed similar expression patterns in response to infection by any two different pathogens (Figure 5C and Supplementary file 2).

Overlapping expression patterns of the *ONAC* genes in response to different abiotic and biotic stresses were analyzed further. In total, 65 *ONAC* genes responsive to abiotic stress and 96 *ONAC* genes responsive to biotic stress were identified. It is noteworthy that 63 *ONAC* genes exhibited overlapping expression patterns in response to abiotic and biotic stresses and most of these *ONAC* genes were upregulated under abiotic (salt, drought, or cold) and biotic (infection by *M. oryzae* and infestation by *S. hermonthica*) stress (Figure 5F). Among these, *ONAC026*, *ONAC069*, *ONAC095*, *ONAC108*, and *ONAC117* were responsive to all the abiotic and biotic stresses, while 8 *ONAC* genes were responsive to most of the abiotic and biotic stresses (Figure 5E, Supplementary file 2).

### 2.4. Evaluation Relationships of ONAC Genes with Overlapping Expression Patterns

**Figure 6:**
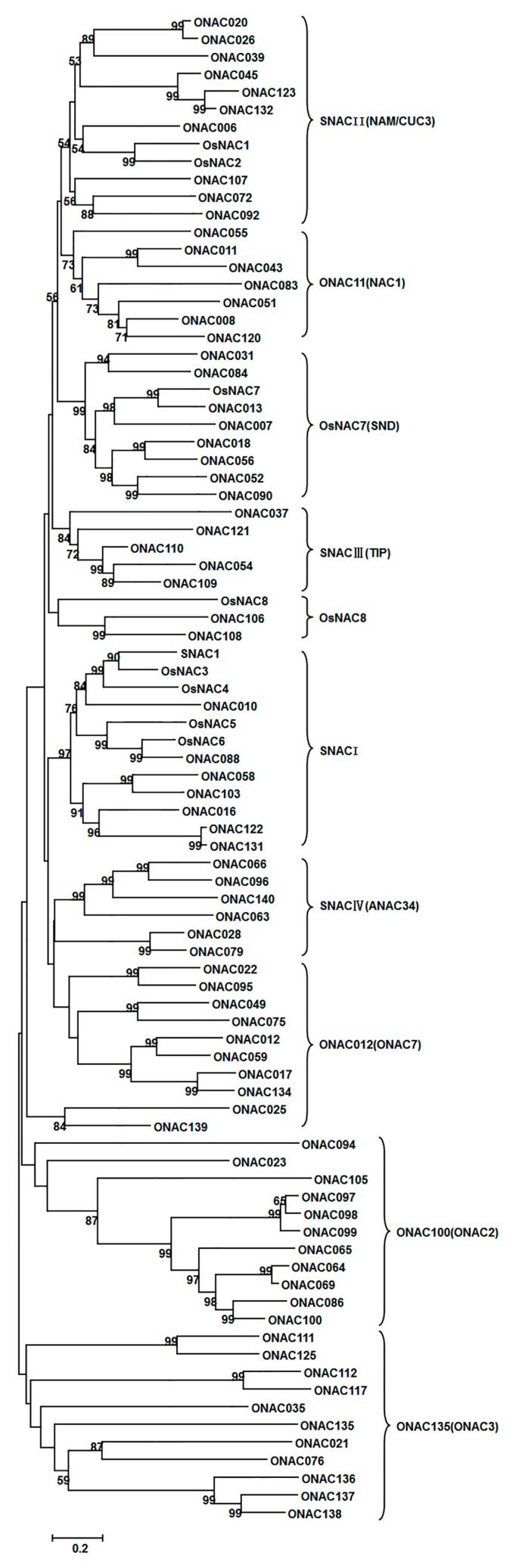
Uprooted phylogenetic tree representing relationships among stress-responsive *ONAC* genes. Full-length protein sequences of 86 *ONAC* genes (63 *ONAC* genes showing overlapping expression patterns, several previously reported *ONAC* genes, and some homologous *ONAC* genes) were used for multiple alignments by the clustalw2 program and a phylogenic tree was created and visualized using MEGA 6.

To clarify possible evaluation relationships, we constructed a phylogenetic tree for a total of 86 *ONAC* genes including 63 *ONAC* genes with overlapping expression patterns, several previously reported *ONAC* genes, and some homologous *ONAC* genes using aligned protein sequences. The resulted phylogenetic tree is similar to the unrooted tree constructed previously by Nuruzzaman *et al.* [15], which showed that the stress-responsive *ONAC* genes can be divided into 10 groups (Figure 6). In our phylogenetic tree, 12 *ONAC* genes belong to the SNACII (NAM/CUC3) group, 7 *ONAC* genes belong to the ONAC11 (NAC1) group, 9 *ONAC* genes belong to the OsNAC7 (SND) group, 5 *ONAC* genes belong to the SNACIII (TIP) group, 3 *ONAC* genes belong to the OsNAC8 group, 12 *ONAC* genes belong to the SNACI group, 6 *ONAC* genes belong to the SNACIV (ANAC34) group, 10 *ONAC* genes belong to the ONAC012 (ONAC7) group, 11 *ONAC* genes belong to the ONAC100 (ONAC2) group, and 11 to the ONAC135 (ONAC3) group. These data suggested that these putative stress-responsive *ONAC* genes show significant evolutional diversity and those *ONAC* genes clustered in same group may have the same or similar biological functions. The SNACI group includes 12 known *ONAC* genes—*SNAC1*, *OsNAC3*, *OsNAC4*, *OsNAC5*, *OsNAC6*, *ONAC010*, *ONAC016*, *ONAC058*, *ONAC088*, *ONAC103*, *ONAC122*, and *ONAC131*—and most members of this group are responsive to different abiotic (e.g., salt, drought, and cold) and biotic (e.g., infection by *M. oryzae* and infestation by *S. hermonthica*) stresses (Figure 5 and Figure 6 and Supplementary file 2). Particularly, 4 of them—*ONAC058*, *ONAC103*, *ONAC122*, and *ONAC131*—were previously shown to be upregulated after infection by viral pathogens, RSV, or rice tungro spherical virus (RTSV) [15]. In the SNACII (NAM/CUC3) group, overexpression of *ONAC045* in rice can improve drought and salt tolerance [31] and *OsNAC1* and *OsNAC2* were identified previously [58]. Importantly, 7 *ONAC* genes—*ONAC026*, *ONAC039*, *ONAC045*, *ONAC092*, *ONAC107*, *ONAC123*, and *ONAC132*—showed upregulated expression patterns in response to abiotic (e.g., salt, drought, and cold) and biotic (infection by *M. oryzae* or RSV) stresses (Figure 5 and Figure 6 and Supplementary file 2). In the SNACIII (TIP) group, *RIM 1* (*ONAC054*) has been demonstrated to be a negative regulator of defense response against rice dwarf virus [41]. *RIM1*, *ONAC110*, and *ONAC121* exhibited upregulated expression patterns under abiotic (e.g., salt, drought, and cold) and biotic (infection by *M. oryzae*) stresses, whereas *ONAC109* showed downregulated expression under all the abovementioned stresses except RSV infection (Figure 5 and Figure 6 and Supplementary file 2). In the SNAC IV (*ANAC34*) group, *ONAC063* was shown to be inducible to high salt stress and overexpression in Arabidopsis increased salt tolerance [59]. *ONAC96* and *ONAC140* exhibited upregulated expression patterns under abiotic (e.g., salt, drought, and cold) and biotic (infection by *M. oryzae* or RSV) stresses (Figure 5 and Figure 6 and Supplementary file 2). *OsNAC8* is highly homologous to Arabidopsis *NTL6*, which has been shown to be a cold-responsive *NAC* gene with function in disease resistance response [60]. *OsNAC8* exhibited upregulated expression patterns under abiotic (e.g., salt, drought, and cold) and biotic (infection by *M. oryzae*) stresses. Most of the *ONAC* genes, such as *ONAC056*, *ONAC052*, and*ONAC084* in the *OsNAC7* (*SND*) group, were downregulated under salt and drought stresses (Figure 5 and Figure 6 and Supplementary file 2), but were upregulated under cold and infection by RSV, *Xoo*, or *Xoc* (Figure 5 and Figure 6 and Supplementary file 2). Expression of *ONAC008* and *ONAC043* in the *ONAC011* (*NAC1*) group was upregulated in response to salt, drought, and cold and to infection by *M. oryzae*, whereas expression of *ONAC043* of this group was induced by *Xoo* and *Xoc* (Figure 5 and Figure 6 and Supplementary file 2). *ONAC028* and *ONAC108*, two members of the *ONAC079* (*ONAC6*) group, were upregulated in response to salt and drought stress and to *Xoc* infection (Figure 5 and Figure 6 and Supplementary file 2). *ONAC012*, *ONAC059*, and *ONAC017* in the ONAC012 (ONAC7) group were induced by cold and *M. oryzae*, whereas *ONAC012* and *ONAC017* were induced by *Xoo* and *Xoc* (Figure 5 and Figure 6 and Supplementary file 2). Most of the members in the ONAC135 (ONAC3) and ONAC100 (ONAC2) groups were responsive to biotic stresses (infection by *M. oryzae*, *Xoo*, *Xoc*, or RSV) (Figure 5 and Figure 6 and Supplementary file 2), indicating their possible involvement in biotic stress response.

## 3. Discussion

In plants, the expression of stress-responsive genes is transcriptionally activated by different forms of abiotic stresses and by different pathogens. Overlapping expression of the stress-responsive genes is a result of coordination of plant responses to different abiotic and biotic stresses [61,62]. In the present study, we identified a total of 63 *ONAC* genes that showed overlapping expression patterns in rice seedlings/plants under different abiotic and biotic stresses from analyses of public microarray data. Most of these *ONAC* genes with overlapping expression patterns (e.g., those belonging to the SNACI, SNACII, SNACIII, SNACIV, and ONAC011 groups) were upregulated in response to abiotic (salt, dry, and cold) and biotic (infection by *M. oryzae* and infestation by *S. hermonthica*) stresses. Among the 63 *ONAC* genes with overlapping expression patterns identified in our analysis, 7 *ONAC* genes—*SNAC1*, *OsNAC3*, *OsNAC4*, *OsNAC5*, *OsNAC6*, *OsNAC045*, and *OsNAC10* (*ONAC122*)—were previously reported to be induced by drought, low temperature, or high salinity conditions [25,26,27,28,29,30,31], indicating the validity of our microarray data-based analysis of the *ONAC* gene expression under different abiotic and biotic stress. Our quantitative RT-PCR analysis of 30 selected *ONAC* genes in rice seedlings after treatments with drought, salt, or cold stresses, or infection by *M*. *oryzae* or RSV, further confirmed the overlapping expression features of these *ONAC* genes, showing similar or identical expression patterns to those obtained from analysis of the rice microarray data. ABA is a well-known stress hormone that mediates signaling pathways involved in defense response against abiotic and biotic stresses [50,51]. In the present study, 16 *ONAC* genes were upregulated in response to ABA. Previous studies have shown that *SNAC1*, *OsNAC5*, *OsNAC6*, *ONAC045*, and *OsNAC10* were strongly induced by ABA [25,26,29,30,31]. These results indicate that these stress-responsive *ONAC* genes might be involved in ABA hormone-mediated signaling pathways and thus play important roles in the stress response. Taken together, members of the ONAC family are responsive to multiple abiotic and biotic stresses and thus might play important roles in mediating defense response against different abiotic and biotic stresses. However, functional analysis using loss-of-function and gain-of-function mutants or transgenic lines is critical to elucidate the biological function of these *ONAC* genes with overlapping expression patterns in rice abiotic and biotic stress response.

Most of the *ONAC* members identified in the present study were shown to be involved in responses to multiple stresses and stress-related signal molecule, and exhibited complicated overlapping expression patterns under different abiotic and biotic stress conditions. Previous studies have shown that members of the *ONAC* genes may have pleiotropic function in tolerance against different abiotic stresses and defense response against biotic stress. For example, transgenic rice plants overexpressing *OsNAC6* exhibited an improved tolerance to dehydration and high-salt stresses, and also exhibited increased tolerance to blast disease [26,28]. Similarly, overexpression of stress-responsive *SNAC1* or *OsNAC5* enhanced drought and salt tolerance in rice and many stress-responsive genes were upregulated in the transgenic plants [25,29]. The responsiveness of these stress-responsive *ONAC* genes with overlapping expression patterns under different abiotic and biotic stress conditions might imply that they have pleiotropic biological functions in the stress response, although detailed functional analysis using mutants and/or overexpression transgenic lines is required. The pleiotropic feature of the biological functions of the *ONAC* genes makes them excellent candidates for efforts aimed at genetic improvement of stress tolerance in various kinds of plants including rice.

## 4. Methods

### 4.1. Plant Growth and Treatments

Rice (*Oryza sativa* L.) subsp. *indica* cultivar Yuanfengzao was used in this study. Seeds were germinated and grown on soil (clay mixed with one-quarter soil) in a greenhouse with natural sunlight. Seedlings were moved to a growth chamber (28 °C 14 h light >1000 μmol·m^−2^·s^−1^/26 °C 10 h dark) 3 days before the experiment.

For ABA treatment, 7-day-old seedlings were sprayed with 100 µM ABA and kept for 3 h. Salt stress was administered by transferring 7-day-old seedlings to a beaker containing 200 mM NaCl solution for 3 h at 28 °C; drought stress was carried out by placing 7-day-old seedlings onto paper towels for 3 h at 28 °C; cold treatment was achieved by keeping 7-day-old seedlings of cv. Yuanfengzao at 4 °C for 3 h. Seedlings kept at 28 °C for 3 h were used as a control. Samples were harvested after designated time points and stored at −80 °C until use.

For inoculation with *M. oryzae*, 2-week-old cv. Yuanfengzao seedlings were inoculated by foliar spraying with spore suspension (5 × 10^5^ spores/ml in 0.02% (*v*/*v*) Tween-20) of strain 85-14B1 of the blast fungus (race ZB1) until uniformly covered with tiny droplets. Mock-inoculation controls were prepared by spraying with 0.02% (*v*/*v*) Tween-20. The inoculated and mock-inoculated seedlings were kept at 100% relative humidity in the dark for 24 h and then returned to the growth chamber. Leaf samples were collected at different time points (72 and 96 h after inoculation) and stored at −80 °C until use. For inoculation with RSV, 9-day-old seedlings were infested by RSV-carrying *Laodelphax striatellus* insects for 2 days and then transferred to an insect-free chamber at 25 ± 3 °C with a daily photoperiod. Control plants were not infested by insects but transferred to the same insect-free chamber. Leaf samples were harvested 8 days after infestation and stored at −80 °C until use.

### 4.2. Mining and Analysis of Microarray-Based Expression Profiling Data

Microarray data publicly available on the Rice Oligo Array Database [63] were used in this study. Data from accessions GSE6901 (expression data for abiotic stress treatment), GSE7256 and GSE18361 (expression data for rice leaf and root infection with *M. oryzae* strains FR13 and Guy11, respectively), GSE16793 (expression data for interactions with *Xoo* or *Xoc*), GSE11025 (expression data for interaction with RSV), and GSE10373 (expression data for interaction with *S. hermonthica*) were mined and analyzed for expression patterns of the rice *ONAC* genes. Information on the *ONAC* genes and entire microarray experiments used in this study is listed in Supplementary file 4, 6 and 7 [49,52,53,55,56,57]. Probe sets for a total of 107 *ONAC* genes were included in the Affymetrix rice genome array and IDs of probe sets representing *ONAC* genes were identified. The probe set of every representative *ONAC* gene (Supplementary file 5) shows normalized signal intensities. Expression data for these 107 *ONAC* genes under different abiotic and biotic stress conditions were mined from the Rice Oligonucleotide Array database, and transferred as log_2_^signal intensity^ value for each probe ID corresponding to *ONAC* genes. A differentially expressed gene in a given abiotic or biotic stress was defined according to the value of log_2_^(signal intensity in treatment/signal intensity in control)^ = log_2_^signal intensity in treatment^ − log_2_^signal intensity in control^, and upregulation or downregulation of *ONAC* genes was chosen for the analysis when the value of log_2_^(signal intensity in treatment/signal intensity in control)^ is >1 or <−1 [15] and the relative expression folds is >2 or <0.5, respectively. The FDR level for detecting significant differential expression was set to 0.01. Genes with a *p*-value of <0.05 were considered to be differentially expressed. Hierarchical clustering heat maps were generated by TM4 MultiExperiment Viewer (MeV), which calculates pairwise Euclidean distances using the average linkage method.

### 4.3. qPCR Analysis

For quantitative real-time PCR analysis, total RNA was extracted from frozen leaf samples with TRIzol (Invitrogen, Shanghai, China), according to the manufacturer’s instructions. Resulting RNA samples were treated with RNase-free DNase (TaKaRa, Dalian, China) and first strand cDNAs were synthesized using AMV reverse transcriptase (TaKaRa, Dalian, China), following the manufacturer’s instructions. QPCR reactions were performed as previously described [64]. Gene specific primers were synthesized for 30 *ONAC* genes and one actin gene (Os05g36290) and listed in Supplementary file 8. Expression data for each *ONAC* gene under different abiotic and biotic conditions were normalized using the expression data of the actin gene as an internal reference. Relative expression level was calculated by the comparative ΔΔ*C*_t_ method [65]. The Δ*C*_t_ and ΔΔ*C*_t_ were calculated by the formulas: Δ*C*_t_(target gene) = *C*_t_(target gene) − *C*_t_(actin) and ΔΔ*C*_t_ = Δ*C*_t_(different time points) − Δ*C*_t_(0 h) or ΔΔ*C*_t_ = Δ*C*_t_(treated sample) − Δ*C*_t_(untreated sample). Expression folds for each *ONAC* gene under a given stress were calculated by the formulas of fold = 2^−ΔΔ*C*t^. Standard errors of the means from three independent biological replicates were calculated.

### 4.4. Phylogenetic Analysis

Protein sequences for 86 *ONAC* genes (63 *ONAC* genes with overlapping expression patterns, several previously reported *OsNAC* genes, and some *ONAC* homologous genes, Supplementary file 3) were obtained from the Rice Genome Annotation [66] and were subject to phylogenetic analysis. The alignment was adjusted manually and unrooted phylogenetic trees were constructed by the neighbor-joining method using MEGA 6 software [67]. The confidence level of monophyletic groups was estimated using a bootstrap analysis of 1000 replicates. A phylogenetic tree was created, and visualized using MEGA 6 software.

## 5. Conclusions

In the present study, we analyzed the expression of *ONAC* genes based on the rice microarray data and the results showed that a total of 63 *ONAC* genes showed overlapping expression patterns in rice seedlings/plants under various different abiotic and biotic stress conditions. These *ONAC* genes with overlapping expression patterns might have pleiotropic biological functions in stress response. Results from this systematic analysis of the *ONAC* gene family presented not only evidence for the possible role of *ONAC* genes in response to abiotic and biotic stresses, but also provided clues for further functional analysis of *ONAC* genes in stress tolerance and pathogen resistance.

## Supplementary Materials

Supplementary materials can be found at http://www.mdpi.com/1422-0067/16/02/4306/s1.
